# Metallothioneins and Megalin Expression Profiling in Premalignant and Malignant Oral Squamous Epithelial Lesions

**DOI:** 10.3390/cancers13184530

**Published:** 2021-09-09

**Authors:** Ana Zulijani, Andrea Dekanić, Tomislav Ćabov, Hrvoje Jakovac

**Affiliations:** 1 Department of Oral Surgery, Clinical Hospital Center Rijeka, Krešimirova ul. 40, 51000 Rijeka, Croatia; ana.zulijani@sz.uniri.hr; 2 Department of Pathology, Clinical Hospital Center Rijeka, Krešimirova ul. 42, 51000 Rijeka, Croatia; andrea.dekanic@medri.uniri.hr; 3 Faculty of Dental Medicine, University of Rijeka, Krešimirova ul. 40, 51000 Rijeka, Croatia; 4 Department of Physiology and Immunology, Faculty of Medicine, University of Rijeka, Ul. Braće Branchetta 20, 51000 Rijeka, Croatia

**Keywords:** metallothioneins, megalin, interaction, oral squamous cell carcinoma, oral leukoplakia, lichen planus, regulated intramembrane proteolysis

## Abstract

**Simple Summary:**

Interactions of metallothioneins with the multiligand receptor megalin have been found to promote cell survival and proliferation after noxious stimuli in non-malignant tissues. However, the relationship between these proteins and their interaction remains to be addressed in malignancies and premalignant lesions. In this retrospective study, we compared the expression profiles of metallothioneins and megalin in different histological grades of oral squamous cell carcinoma, oral leukoplakia, and oral lichen planus. The data obtained show a gradual increase of both proteins concomitantly with the gaining of more malignant features, as well as their co-expression and direct interaction in carcinomatous tissue. The results point to the pro-oncogenic role of the metallothionein-megalin functional axis, which may impact tumor phenotype and behavior.

**Abstract:**

This study aimed to assess the relationship and possible interactions between metallothioneins (MTs) and megalin (LRP-2) in different grades of oral squamous cell carcinoma (OSCC) and premalignant lesions of the oral mucosa (oral leukoplakia and oral lichen planus). The study included archived samples of 114 patients and control subjects. Protein expression was examined by immunohistochemistry and immunofluorescence, and staining quantification was performed by ImageJ software. Protein interaction in cancer tissue was tested and visualized by proximity ligation assay. Mann-Whitney and Kruskal-Wallis tests were used to determine the significance of differences between each group, whereas Pearson correlation coefficient was performed to test correlation. Expression of both proteins differed significantly between each group showing the same pattern of gradual increasing from oral lichen planus to poorly differentiated OSCC. Moreover, MTs and megalin were found to co-express and interact in cancer tissue, and their expression positively correlated within the overall study group. Findings of prominent nuclear and chromosomal megalin expression suggest that it undergoes regulated intramembrane proteolysis upon MTs binding, indicating its ability to directly affect gene expression and cellular division in cancer tissue. The data obtained point to the onco-driving potential of MTs-megalin interaction.

## 1. Introduction

Metallothioneins (MTs) are a phylogenetically conserved family of low molecular weight (6–7 kDa) single-chained and cysteine-rich proteins [1,2,3]. In humans, there are four major isoforms of MTs, encoded by clustered genes at chromosome 16q12-22 and identified as MT-I to MT-IV [1]. MT-I and MT-II show a high degree of sequence matching and are commonly co-regulated, being considered as one functional unit denoted as MT I/II and usually referred as MTs in the narrow sense [2]. The main functional features of MTs emanate from their structural properties. The abundance of thiolate (sulfhydryl) groups arranged in clusters enables MTs to bind physiological (Zn, Cu) and toxic (Cd, Hg) heavy metal ions with high affinity [4,5]. Besides, the thiolate cluster can be easily oxidized [6], whereby metal ions are released, becoming available to metal-dependent enzymes and transcription factors [7]. Due to those peculiarities, MTs are involved in toxic metal detoxification [5] and act as a potent endogenous antioxidant system [6], providing protection from oxidative stress that is a common denominator of a multitude of noxious stimuli and an important mediator of the secondary pathophysiological processes [4,8,9,10]. Nevertheless, since MTs pose an interchangeable pool of zinc ions that can be further transferred or brought back to be bound, their expression can modulate the activity of numerous cellular enzymes, signaling proteins and transcription factors dependent on zinc availability [7,11]. Owing to these effects, MTs are implicated in the regulation of basic cellular processes, such as cellular growth, differentiation, proliferation, and apoptosis [4,12]. Increased MTs expression has been found to favor cell survival and multiplication, whereby suppresses apoptotic processes, either by directly modulating zinc-dependent transcription factors such as p53 and nuclear factor κB (NFκB) or by protecting DNA from oxidative damage [1,3,13,14,15,16,17,18]. Exerting such cytoprotective and genoprotective activities, MTs ordinarily translocate from cytoplasm to the cellular nucleus [19,20]. Although MTs were being considered for decades as a strictly intracellular protein, recent studies have shown their active secretion into the extracellular environment, but the executive mechanism remained unclarified [21,22]. More recently, several studies indicated that extracellular MTs can bind to multiligand scavenger receptor megalin, also known as low-density lipoprotein receptor-related protein-2 (LRP-2) and gp330 [23,24]. It is thought that resulting membrane complex MT-megalin could either trigger pro-survival and pro-mitotic pathways via protein kinase B (PKB, AKT-1) activation through SH2/SH3 domains or/and undergo endocytic internalization regulated by NPXY sequences, thereby supplying the cell with “exogenous” MTs, that may be even directly shuttled into the nucleus upon endocytosis [25,26,27,28,29]. Apart from that, upon ligand binding, megalin can be, as with Notch, subjected to regulated intramembrane proteolysis (RIP) resulting in cleavage and release of C-terminal soluble megalin intracellular domain (LRP-2-ICD; MICD), which consequently enters the cell nucleus where directly modifies gene expression [30,31,32]. Data on the paracrine MT-megalin axis are scarce and mostly limited to the central nervous system (CNS), where such interactions were found to be associated with neuronal survival, neuroprotection and neuroregeneration [24,26,28,29,30]. In the view of this knowledge, particular interest was drawn to the possible role of MTs in the processes of oncogenesis and tumor progression, and MTs expression was being extensively investigated on that trail [1,3,33,34,35]. However, despite the large corpus of data in this field, they are inconsistent and do not allow convincing conclusions on MTs impact on carcinogenesis, failing to firmly establish these molecules as a reliable diagnostic and prognostic marker of oncological diseases [33,34]. The heterogeneity of the data collected so far suggests that MTs expression in tumorous tissue is related to tumor type and origin, differentiation status and histological grade, which all can affect tumor aggressiveness and prognosis [34]. Furthermore, MTs expression also could depend on the presence and type of exogenous carcinogenic stimuli, since many carcinogenic environmental factors, such as chronic intoxication, smoking and radiation, have been found to induce MTs in nonmalignant tissues [36,37,38,39]. At this point, it is worth mentioning that the expression of MTs is considered to be an adaptive response in healthy tissues, providing protection from oxidative DNA damage, thereby reducing the probability of gaining carcinogenic mutations [1,19,39,40]. Considering these effects, the impact of MTs during oncogenesis and cancer progression could be proposed as a “double-edged sword”, protecting premalignant lesions from malignant transformation, but allowing unhindered and rapid cell cycling in already malignant transformed lesions [1]. Furthermore, the behavior and fate of both premalignant and malignant cells exposed to extracellular MTs, which may be either actively secreted from surrounding cells or passively released from injured or necrotic tissue, can be influenced by the concomitant expression of megalin receptor that further amplify MTs pro-survival effects. Importantly, some megalin ligands, such as transthyretin, have been found to enhance megalin expression by positive feedback mechanism triggered upon megalin RIP processing and LRP-2-ICD nuclear translocation [30]. It is noteworthy that the RIP process requires metalloproteinases activity [30,31,41] which considerably depends on available Zn^+^ ions [42], which in turn may be ensured by transferring from MTs [42], serving thus as a permissive factor for LRP-2-ICD release. However, megalin expression in cancer tissue and premalignant lesions remains understudied, and varying expression of megalin might be a clue to the inconsistent results on MTs relation to cancer aggressiveness, obtained when MTs expression was considered separately [34]. Aiming to expand still limited data on this topic and to complement our recent findings regarding uterine cervical squamous lesions [43], in the present study we analyzed, compared, and correlated expression profiles of MT I/II and megalin in different histological grades of oral squamous cell carcinoma (OSCC), as well as in oral lichen planus (OLP) and oral leukoplakia (OL) as the most common and well histologically defined oral potentially malignant disorders (OPMD) progressing in some cases to OSCC [44,45]. Assuming the possible interactions, we also assayed MTs/megalin co-expression and their ligation in carcinomatous tissue. In addition, the effect of smoking habit, sex, and age on the expression of both proteins were assessed.

## 2. Materials and Methods

### 2.1. Patients and Specimens

The study included a total of 114 subjects diagnosed with OLP (N = 15), OL (N = 26), or OSCC (N = 63), as well as 10 healthy volunteers served as a control group with healthy oral mucosa (HOM). The OSCC group comprised 28 patients diagnosed with grade I cancer, 23 with grade II, and 12 with grade III. The sample size for each group was calculated using the open-source sample size calculator Sampsize (Sourceforge; https://sourceforge.net/projects/sampsize/ (accessed on 3 June 2020)) with an accuracy of 5% and confidence interval of 95%. The inclusion criteria were the clinical and histological established diagnosis of the respective oral lesion. Exclusion criteria for each group were presence of any other acute/chronic oral mucosal pathology or systemic disease, as well as undergoing chemotherapy or radiotherapy prior to the tissue sampling. The average age of patients was 62.8 ± 10.1 years (range 24–86; median age 63 years). 37% (N = 42) of enrolled patients were female, and 63% (N = 72) were male. Basic demographic and clinicopathological characteristics of involved subjects are given in Table 1. Metallothionein I/II and megalin expressions were assessed immunohistochemically on randomly selected samples from tissue collection archived at Clinical Department of Pathology and Cytology, Clinical Hospital Center, Rijeka. Diagnoses of all tissue samples were confirmed by two independent pathologists and classified according to the 4th edition of the World Health Organization (WHO) [46] and the 8th edition of the AJCC Cancer Staging Manual [47]. Briefly, histological grading of OSCC samples was based on the histological architecture and tumor cell differentiation (morphological similarity to the healthy oral mucosa and degree of keratinization), nuclear atypia and presence/location of mitotic figures, as follows: grade I—easily recognizable squamous epithelium, clear evidence of stratification with basal cell layer surrounding the well-defined tumorous islets, abundant central keratinization forming “keratin pearls”, minimal pleomorphism, only basally located mitotic figures; grade II—moderately differentiated tumorous cells, focal keratinization, features between grade I and grade III; grade III—poorly differentiated and pleomorphic tumorous cells with considerable nuclear atypia, no or minimal keratinization, difficult to identify squamous epithelial origin of tumorous tissue, diffusely prominent mitotic figures that are often abnormal in appearance. Regarding the localization of the lesions, 27 specimens originated from the anterior two-thirds of the tongue (OLP-2, OL- 7, OSCC grade I-9, OSCC grade II-5, OSCC grade III-4), 23 from the floor of the oral cavity (OLP-2, OL- 2, OSCC grade I-7, OSCC grade II-8, OSCC grade III-4), 22 from the buccal mucosa (OLP-9, OL- 6, OSCC grade I-5, OSCC grade II-1, OSCC grade III-1), 23 from the gingiva (OLP-2, OL- 8, OSCC grade I-6, OSCC grade II-5, OSCC grade III-2), and 9 from retromolar area (OLP-0, OL- 3, OSCC grade I-1, OSCC grade II-4, OSCC grade III-1). Clinical data were retrieved retrospectively using the national hospital information system. Samples of the healthy oral mucosa were collected at the Clinic of Dental medicine, Clinical Hospital Center in Rijeka during the surgical removal of the impacted third molars, frenectomies, and open corticotomies (6 specimens originated from the gingiva and 4 from retromolar area).

### 2.2. Immunohistochemistry

Immunohistochemistry was performed on paraffin-embedded tissue sections sliced to a thickness of 4 µm using rotary microtome HM 340 E (Microm/Thermo Fisher Scientific, Walldorf, Germany). Slides were dewaxed by Tissue Clear (Sakura Finetek Europe, The Netherlands) and rehydrated in graded ethanol solutions. Before the staining procedure, antigen demasking was applied by heating tissue sections in 10-mM and pH 6.0 sodium citrate buffer. Staining was performed using highly sensitive and specific DAKO EnVision+System, Peroxidase (DAB) kit (DAKO Cytomation, Santa Clara, CA, USA) as previously described [43]. Briefly, after blockage of non-specific binding and endogenous peroxidase activity with commercial blocking solution (DAKO Cytomation, Santa Clara, CA, USA), slides were incubated with mouse monoclonal anti-MT I + II antibody (clone E9; Dako Cytomation, Santa Clara, CA, USA; diluted 1:100 with 1% BSA in PBS) or rabbit polyclonal anti-megalin antibody (H-245, Santa Cruz Biotechnology, Dallas, TX, USA; diluted 1:200 with 1% BSA in PBS) for 12 h at 4 °C in a humid chamber. After rinsing, tissue sections were incubated with peroxidase-labeled polymer linked to secondary goat anti-mouse and anti-rabbit antibodies for 30 min at room temperature and humid environment. Immunoreactions were rendered visible using peroxidase chromogenic substrate DAB (3,3′-Diaminobenzidine). Tissue samples were afterward counterstained by hematoxylin immersion for 30 s, dehydrated in an ethanol gradient, and mounted with Entellan (Sigma-Aldrich, Hamburg, Germany). Immunohistochemical staining by means of both previously declared primary antibodies may result in cytoplasmic, nuclear, and membrane immunoreactivity. To verify specificities of immunohistochemical reactions, primary antibodies were substituted with isotype-matched control antibodies (monoclonal mouse IgG, clone DAK-G01, DAKO Cytomation, Santa Clara, CA, USA; polyclonal rabbit IgG, Abcam, UK) used in the same conditions and dilutions on negative control slides. All slides that served as negative control showed no immunohistochemical signals. Tissues were examined by the Olympus BX51 microscope and (Olympus, Tokyo, Japan), and photomicrographs were taken with a DP50 camera system using Cell ^F software (Olympus, Tokyo, Japan).

### 2.3. Immunohistochemical Staining Quantification

Quantification of immunohistochemical staining was performed on captured photomicrographs using open-source ImageJ software. Staining resulted from the peroxidase-DAB reaction was separated and segmented by the color deconvolution plug-in. The separated immunohistochemical signals were then converted to 16-bit images comprising different gray values that vary proportionally to the intensity of the immunohistochemical staining. The numerical data obtained from each image reflected integrated gray intensities of each particular cell per area covered by the microscopic field at × 400 magnification, thereby rendering the average gray value used for further statistical analyzes. The average gray value was measured for each case analyzing five randomly selected microscopic fields per slide of a tissue sample. Data were expressed in arbitrary units (AU) as the median gray value of each group with range.

### 2.4. Immunofluorescence

Double immunofluorescence labeling was carried out on dewaxed and rehydrated tissue sections that had been treated for antigen retrieval as described above. One-hour incubation with a blocking solution (1% BSA and 0.001% NaN3 in PBS) at room temperature was used to prevent nonspecific antibody binding. Tissues were subsequently incubated with mouse monoclonal anti-MT I + II antibody (clone E9; Dako Cytomation, Santa Clara, CA, USA; diluted 1:50 with blocking solution) and rabbit polyclonal anti-megalin antibody (H-245, Santa Cruz Biotechnology, Dallas, TX, USA; diluted 1:50 with blocking solution) for 12 h at 4 °C in a humid chamber. After being rinsed with PBS, tissues were incubated with goat anti-mouse IgG secondary antibody conjugated with Alexa Fluor 555 (Thermo Fisher Scientific, Waltham, MA, USA; diluted 1:500 with blocking solution) and donkey anti-rabbit IgG secondary antibody conjugated with Alexa Fluor 488 (Thermo Fisher Scientific, Waltham, MA, USA; diluted 1:300 with blocking solution) for 1 h at room temperature in a humid and dark environment. Visualization of nuclei was achieved by staining with 4′,6-diamidino-2-phenylindole, dihydrochloride (DAPI; Thermo Fisher Scientific, Waltham, MA, USA; diluted 1:1000 with PBS) and slides were eventually mounted with Mowiol (Sigma-Aldrich, Hamburg, Germany). To confirm the specificity of the immunoreactions, negative controls were done by replacing the primary antibodies with isotype-matched control immunoglobulins used under the same conditions. Photomicrographs were captured by the Olympus BX51 fluorescence microscope (Olympus, Tokyo, Japan) equipped with a DP50 camera and Cell ^F software (Olympus, Tokyo, Japan).

### 2.5. Proximity Ligation Assay

Proximity ligation assay (PLA) was performed on tissue sections using Duolink PLA In Situ Fluorescence kit (Sigma-Aldrich, Hamburg, Germany) according to the manufacturer’s protocol and using in-house modified diluent and blocking solutions as previously described [48]. In brief, after rehydration, tissues were incubated with 1% BSA in PBS for 1  h at room temperature to block nonspecific antibody binding, followed by overnight incubation with mouse monoclonal anti-MT I + II (clone E9; Dako Cytomation, Santa Clara, CA, USA; diluted 1:50 with blocking solution) and rabbit polyclonal anti-megalin (H-245, Santa Cruz Biotechnology, Dallas, TX, USA; diluted 1:50 with blocking solution) antibodies at 4  °C in a humid chamber. Following rinsing in PBS, tissues were incubated in a pre-heated humidity chamber for 1 h at +37 °C with anti-mouse PLUS and anti-rabbit MINUS PLA probes having been diluted 1:5 with blocking solution and sited at room temperature for 20  min immediately before adding. Thereafter, slides were washed with 1 × Wash Buffer A (prepared according to enclosed instructions) and the ligation reaction was enabled by incubating slides with Ligation-Ligase solution (Ligation stock diluted 1:5 in nuclease-free water with immediately added ligase at a 1:40 dilution) in a pre-heated humidity chamber for 30 min at +37 °C. After washing in 1 × Wash Buffer A, amplification reactions on samples were performed by incubation with Amplification-Polymerase solution (Amplification stock diluted 1:5 in nuclease-free water with immediately added Polymerase at a 1:80 dilution) in a pre-heated humidity chamber for 100  min in the dark at +37 °C. Tissues were subsequently washed twice in 1 × Wash Buffer B (prepared according to enclosed instructions) for 10  min, and then twice in 0.01 × Wash Buffer B for 1  min, continuously protected from the light. Nuclei were visualized by DAPI staining (Thermo Fisher Scientific, Waltham, MA, USA; diluted 1:1000 with PBS) and Mowiol (Sigma–Aldrich, Hamburg, Germany) was used as a mounting medium. The images were taken under a fluorescent microscope using a DP50 camera (Olympus, Tokyo, Japan).

### 2.6. Statistical Analysis

Statistical analyses were performed using Statistics software version 12 (StatSoft Inc., Tulsa, OK, USA). The Kolmogorov-Smirnov test was used to assess the normality of the data distribution. The Mann-Whitney test was used to examine differences between the central tendencies of the two groups. Differences between groups were assessed using the Kruskal-Wallis test with post-hoc analysis according to Conover. Correlations were tested by the Pearson correlation coefficient. Statistical significance was set at *p* < 0.05. All quantitative data are presented as a median value with range.

### 2.7. Ethical Statement

The Ethics Committee of Medical Faculty in Rijeka (protocol code: 003-08/20-01/85, number: 2170-24-09-8-20-3, 01.09.2020.) and Ethics Committee of Clinical Hospital Center in Rijeka (protocol code: 003-05/19-1/121, number: 2170-29-02/1-19-2, 24.09.2019) approved this study. The study complied with all ethical standards and guidelines of the Helsinki Declaration.

## 3. Results

### 3.1. Metallothionein I/II Expression in OLP, OL and Different Grades of OSCC

**Figure 1:**
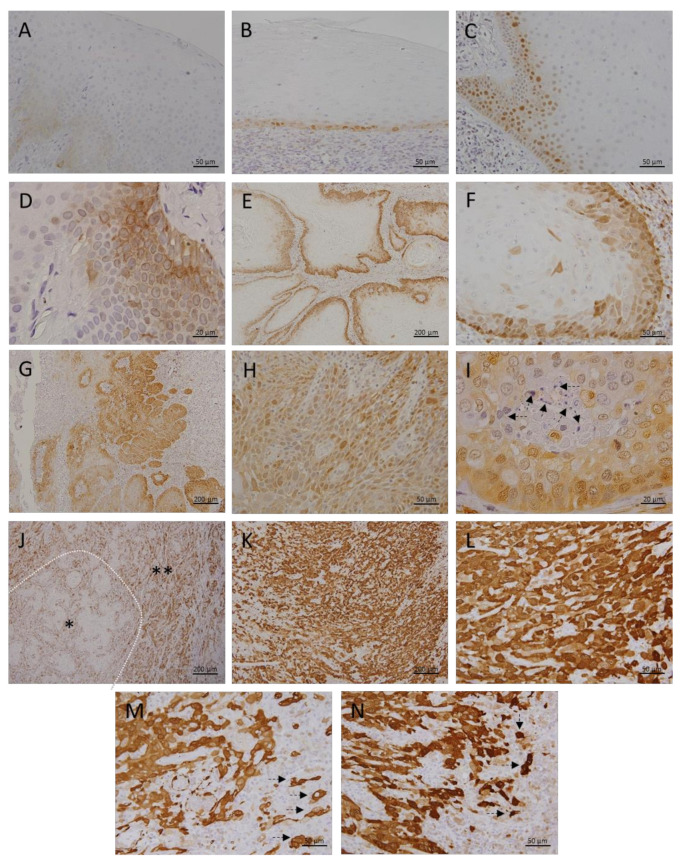
Metallothionein I/II expression in premalignant and malignant oral squamous epithelial lesions. Representative photomicrographs show immunohistochemical staining with anti-MT I/II antibody on paraffin-embedded sections of tissue samples obtained from subjects with healthy oral mucosa (**A**), patients with oral lichen planus (**B**), oral leukoplakia (**C**,**D**), grade I OSCC (**E**,**F**), grade II OSCC (**G**–**I**), mixed-grade (I/II) OSCC (**J**), and grade III OSCC (**K**–**N**). Arrows on I indicate pyknotic nuclei and apoptotic bodies in areas lacking MT I/II immunopositivity. Asterisks on J mark grade I (*) and grade II (**) cancer tissue in mixed-grade tumor. Arrows on M and N point to MT I/II immunopositive cancer cells infiltrating adjacent non-immunopositive healthy tissue at tumor edges. Magnifications: (**E**,**G**,**J**,**K**) × 100; (**A**–**C**,**F**,**H**,**L**,**M**,**N**) × 400; (**D**,**I**) × 1000.

**Figure 2:**
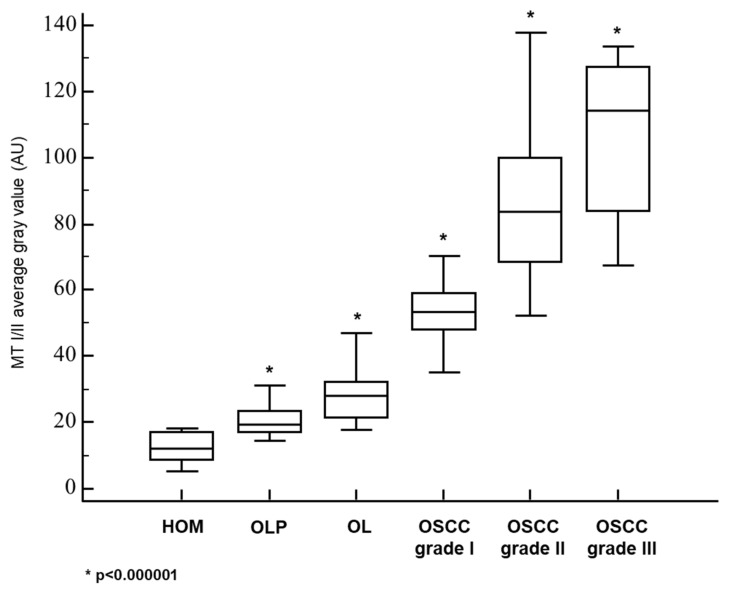
Quantified metallothionein I/II staining intensities in premalignant and malignant oral squamous epithelial lesions. The measurements were made by ImageJ software and data are expressed in arbitrary units (AU) as the median of average gray values with range. HOM, healthy oral mucosa; OLP, oral lichen planus; OSCC, oral squamous cell carcinoma. * *p* < 0.000001 when each group is compared to the other.

In the healthy oral mucosa (HOM), we detected only slight cytoplasmic MT immunopositivity restricted to the basal cell layer (Figure 1A). MT expression was not continuously present along the entire length of the analyzed mucosa, and areas showing a complete absence of MT expression were being frequently found. Mucosa affected by OLP showed more pronounced MT expression, which was found predominantly in the cytoplasm, but also in some nuclei (Figure 1B). Despite MT expression in OLP lesions was also limited to the basal layer, quantification showed significantly higher staining intensity compared with normal mucosa (Figure 2, Table 1). In contrast, OL lesions were found to express MT even in the higher layers of epithelium showing dysplastic changes, wherein cytoplasmic and nuclear staining was observed (Figure 1C). Certain cells in the parabasal layers of OL lesions also showed membrane and perinuclear MT staining (Figure 1D), suggesting intercellular MT trafficking and its intracellular redistribution. Quantitative analysis confirmed significantly higher MT expression in OL relative to OLP (Figure 2, Table 1). In well-differentiated, grade I carcinomas retaining epithelial stratification, considerable MT expression was found in basal and several successive layers surrounding the tumorous islets, whereas MT immunostaining was almost completely absent in central areas comprising cells of squamous morphology and keratin pearls (Figure 1E). However, individual, clearly demarcated MT immunopositive cells with atypical morphology were being frequently observed to have pervaded inner parts of islets (Figure 1F). Grade II OSCC showed diffuse MT immunostaining throughout to all tumorous tissue (Figure 1G), but with different staining intensity of particular cells, emanating thus “mosaic appearance”. Cells with large, pleomorphic nuclei showed generally more intense nuclear staining, while cytoplasmic staining was fairly uniform in all cells (Figure 1H). Described distinctive staining patterns were firmly consistent and clearly distinguishable even within the parenchyma of mixed-grade tumors (Figure 1J). Interestingly, in grade II OSCC samples we observed small, limited areas lacking MT expression but containing cells with pyknotic nuclei and dense, basophilic fragments corresponding to apoptotic bodies (Figure 1I; arrows). In contrast, grade III OSCC exhibited strikingly intense MT staining that was homogeneously widespread over the entire tumor tissue and equally distributed in the nuclei and cytoplasm (Figure 1K,L). At non-cohesive tumor edges, strong MT immunopositivity was also found in the cellular cords and individual cells infiltrating adjacent tissue, highlighting a clear distinction of tumor cells from the non-positive surrounding (Figure 1M,N; arrows). Data obtained by quantitative analysis revealed statistically significant differences in staining intensities between all OSCC grades, as well as in respect to premalignant lesions (Figure 2, Table 1), pointing to the gradual increase in MT expression along with loss of differentiation and gain of higher proliferation potential.

### 3.2. Megalin Expression in OLP, OL and Different Grades of OSCC

**Figure 3:**
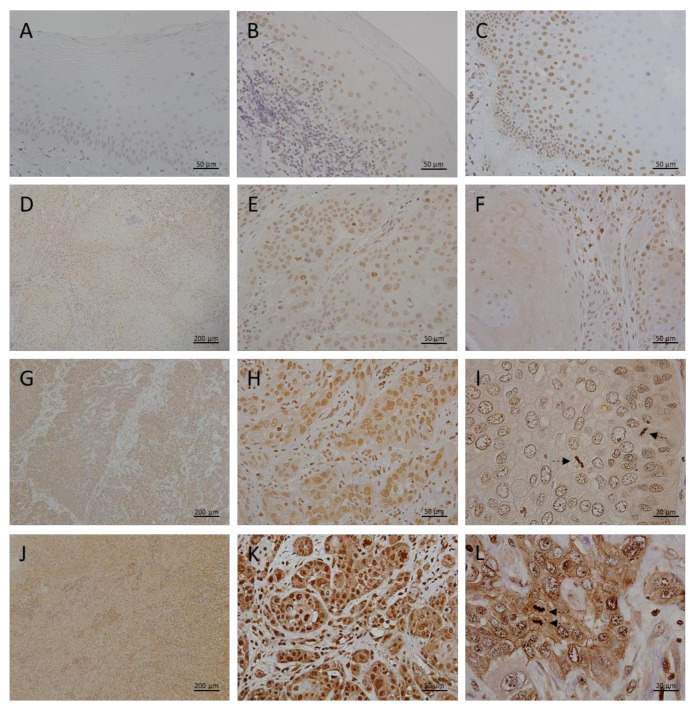
Megalin expression in premalignant and malignant oral squamous epithelial lesions. Representative photomicrographs show immunohistochemical staining with the anti-megalin antibody on paraffin-embedded sections of tissue samples obtained from subjects with healthy oral mucosa (**A**), patients with oral lichen planus (**B**), oral leukoplakia (**C**), grade I OSCC (**D**–**F**), grade II OSCC (**G**–**I**), and grade III OSCC (**J**–**L**). Arrows on I and L indicate chromosomal megalin immunopositivity in mitotic cells. Magnifications: (**D**,**G**,**J**) × 100; (**A**–**C**,**E**,**F**,**H**,**K**) × 400; (**I**,**L**) × 1000.

**Figure 4:**
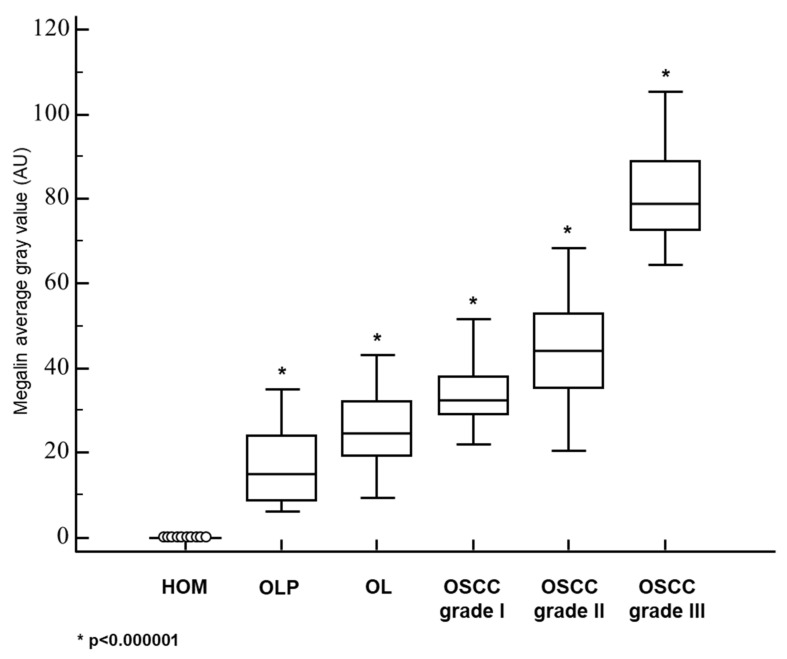
Quantified megalin staining intensities in premalignant and malignant oral squamous epithelial lesions. The measurements were made by ImageJ software and data are expressed in arbitrary units (AU) as the median of average gray values with range. HOM, healthy oral mucosa; OLP, oral lichen planus; OSCC, oral squamous cell carcinoma. * *p* < 0.000001 when each group is compared to the other.

Epithelial cells of healthy oral mucosa did not show immunohistochemically detectable megalin expression (Figure 3A). In OLP lesions, we found only weak megalin expression, restricted to the cells of the basal epithelial layer immediately overlying pathognomonic lympho-histiocytic infiltrates (Figure 3B). Similar to MT, in OL samples megalin was found to have been more extensively expressed in higher epithelial strata showing cellular pleomorphism (Figure 3C), and quantitative analysis showed significantly higher mean staining intensity compared to OLP lesions (Figure 4, Table 1). Grade I OSCC tissues were found to have expressed megalin most prominently in basal and parabasal layers of tumor islets, whereas more central areas showed only weak megalin immunopositivity (Figure 3D–F). Quantitative comparison of average staining intensities showed significantly higher values for grade I OSCC samples when compared to both, OLP and LP (Figure 4, Table 1). Grade II OSCC samples were found with diffuse megalin staining of moderate intensity throughout the tumor tissue (Figure 3G–I). A similar distribution of megalin immunoreactivity was also found in grade III OSCC samples, but the resulting staining was noticeably stronger (Figure 3J–L). In all cancerous tissues, we found cytoplasmic and nuclear megalin immunopositivity, wherein nuclear staining consistently showed a distinctive granular pattern (Figure 3I,L), indicating the possibility that the cleaved MICD domain was transcriptionally active in tumor cells. Moreover, striking megalin immunopositivity was also found in chromosomes of cells showing mitotic figures (Figure 3I,L; arrows), implicating its direct role in the cellular division process. As in the case of MT, quantitative analysis disclosed significant differences in staining intensities, with the highest values for grade III OSCC, and lowest for grade I OSCC samples (Figure 4, Table 1).

### 3.3. Co-Expression and Interaction Studies

**Figure 5:**
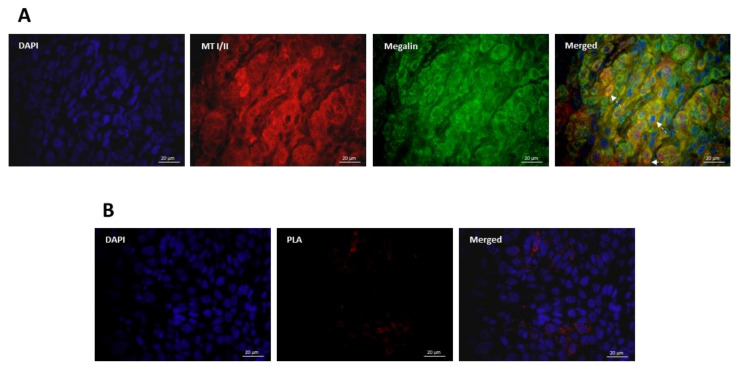
Co-expression and interaction of metallothionein I/II and megalin in oral squamous cell carcinoma. (**A**) Representative photomicrographs of double immunofluorescence staining with anti-MT I/II (red staining) and anti-megalin (green staining) antibodies on paraffin-embedded sections of the OSCC tissue. Blue marks DAPI staining of nuclei. Arrows indicate sites of membrane colocalization. Magnification: × 1000. (**B**) Representative photomicrographs obtained by proximity ligation assay (PLA) on paraffin-embedded sections of the OSCC tissue using anti-MT I/II and anti-megalin antibodies. Red fluorescent signals represent interaction sites. Blue marks DAPI staining of nuclei. Magnification: × 1000.

Since both proteins were found to have been highly induced throughout the OSCC tissues, we further assessed whether there was an overlap in MT I/II and megalin expression in cancer cells. As shown in Figure 5A, data obtained by double immunofluorescence demonstrated widespread co-expression of MT I/II and megalin in carcinomatous tissue. Although in the vast majority of the cells co-expression was present in the cytoplasm and nuclei, particular cells showed strong double-positive membrane signals (Figure 5A, arrows), indicating plasmalemmal MT/megalin co-localization. Aiming to further examine possible interactions of MT I/II and megalin in OSCC tissues, we performed proximity ligation assay (PLA) enabling in situ visualization of protein interaction sites as separate or aggregated punctiform fluorescent signals. The results obtained clearly showed that MT I/II and megalin were not only co-expressed but also did interact in carcinoma tissues (Figure 5B).

### 3.4. Correlation Analysis

**Figure 6:**
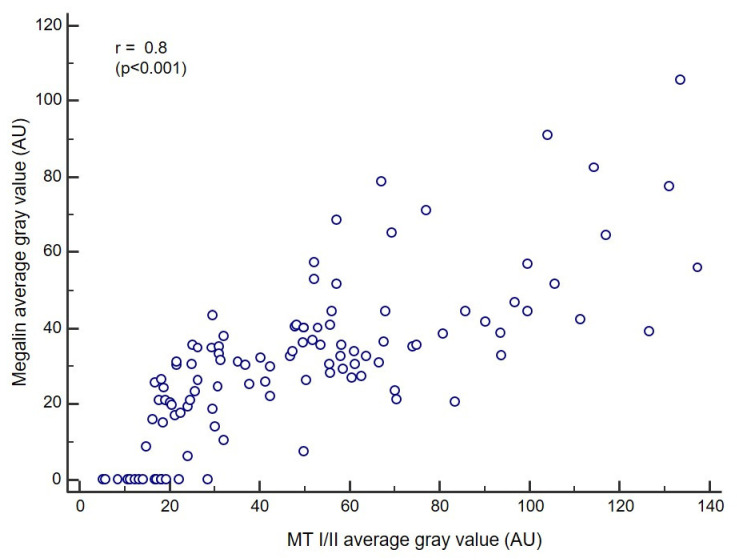
Correlation of metallothionein I/II and megalin staining intensities. Pearson correlation analysis included data obtained from the overall study group. Correlation coefficient (r) = 0.8 (*p* < 0.001).

As differences in staining intensities regarding to a particular diagnosis showed a similar pattern for both, MT I/II and megalin, we assessed whether there was a correlation between the expressions of these proteins. The obtained Pearson correlation coefficient (r) of 0.8 (*p* < 0.001) indicated a strong correlation when the overall study group was included (Figure 6).

### 3.5. Impact of Smoking, Sex, and Age on MT I/II and Megalin Expression

**Figure 7:**
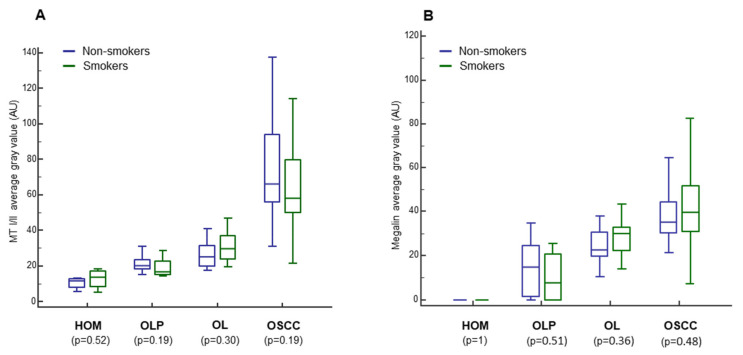
Impact of smoking habit on metallothionein I/II (**A**) and megalin (**B**) expression. The measurements were made by ImageJ software and data are expressed in arbitrary units (AU) as the median of average gray values with range. HOM, healthy oral mucosa; OLP, oral lichen planus; OSCC, oral squamous cell carcinoma.

Since smoking, sex, and age have been found to be involved in OLP, OL and OSCC pathogenesis, we examined the impact of these factors on the staining intensity of MT I/II and megalin for each group separately. However, we found no significant differences neither between smokers and non-smokers (Figure 7) nor between the sexes (data not shown) regarding both proteins analyzed. Nevertheless, correlation analysis using Spearman’s rank-order showed an increase in the expression of MT I/II (r_s_ = 0.3, *p* = 0.0062) and megalin (r_s_ = 0.3, *p* = 0.0005) with age (data not shown).

## 4. Discussion

The results presented confirm the findings of previous studies showing MTs overexpression in OLP [49,50], OL [51,52], and OSCC [53,54,55,56,57,58,59] when each of these lesions was compared with normal oral mucosa. However, in the present study, we systematically analyzed the relationship of MTs expression between premalignant lesions and different grades of OSCC (Figure 1 and Figure 2; Table 1). Results clearly showed a gradual increase of MTs expression from the OLP to poorly differentiated OSCC (Figure 2), indicating that accumulation of these proteins in oral mucosa plays an important role in the gradual process of gaining malignant features during oncogenesis. Nevertheless, the most important novelty of this study is the finding of a concomitant and positively correlated increase in the megalin expression (Figure 3, Figure 4, Figure 5 and Figure 6) which has been found to act as a receptor for extracellular MTs in several types of cells [23]. Despite great attention is recently drawn by the role of extracellular MTs, modes of their release and mechanisms mediating their effects are poorly understood and mostly limited to the CNS pathology [23,24]. The results obtained from this study comply with our recent findings on simultaneous gradual induction of MTs and megalin during the premalignant transformation of squamous epithelia of the uterine cervix [43], but, to the best of our knowledge, here we for the first time showed induction of megalin and its interaction with MTs in malignant carcinomatous tissue of epithelial origin. So far, only a few studies have addressed the attention on the possible role of megalin in tumorigenesis. Pedersen and colleagues [60] found increased expression of megalin and MTs in cerebral lymphomas, which was associated with the presence of oxidative stress markers. Acquisition of megalin expression was also found in the melanoma tissue, wherein the siRNA-induced reduction of megalin synthesis decreased proliferation and survival rates of melanoma cell lines [61]. Additionally, one population-based study linked polymorphisms within the megalin gene with the progression of prostate cancer [62]. Interestingly, megalin mRNA and protein levels were found to decrease in kidney and gallbladder epithelial cell lines upon in vitro activation of transforming growth factor-beta (TGF-ß1)—SMAD2/3 signaling pathway [63], which is well known for its tumor-suppressive effect [64]. As megalin is a multiligand receptor, to clarify its role in tumor growth, attention should be paid to its interaction with possible ligands. On that trail, our findings of megalin and MTs co-expression and their interaction (Figure 5) bring new mechanistic insights on the pro-oncogenic role of both proteins. Apart from previously known pro-survival and anti-apoptotic effects of intracellular MTs, confirmed by our findings of apoptotic tumor cells that “escaped” from MTs induction (Figure 1I), micro-environmental MTs could trigger onco-promoting effects of megalin by binding to it. Our findings of membrane MT immunopositivity on particular dysplastic and carcinoma cells (Figure 1D and Figure 5A) strongly suggest transplasmalemmal trafficking of MTs, which have previously been shown to be able to be actively secreted from the cultured cells [22]. As described before, MTs binding to megalin may result with receptor-ligand complex internalization, thereby supplying cells with additional MTs [25,26,27,28,29], as well as with protein kinase B (AKT-1) activation [25,26,27,28,29], which provide mitogenic signals to the cells being at the same time protected from the apoptotic clearance due to the high MT content. Such a scenario is supported by the studies showing the positive correlation between phosphorylated AKT and MTs expression in OSCC tissue [52] and co-expression of phosphorylated AKT and megalin in squamous epithelia of premalignant uterine cervical lesions [43]. Furthermore, consistent findings of cytoplasmic megalin immunopositivity in our samples (Figure 3) point to rapid endocytic trafficking of megalin, which has previously been described as a fast-recycling and slow-degrading receptor [65]. Similarly, Andersen et al. found that megalin extensively accumulated in Rab5-positive intracellular vesicles of melanoma cell lines [61]. However, more interestingly, here we demonstrated nuclear megalin staining in both premalignant and malignant oral lesions (Figure 3I,L), which strongly suggest that megalin was undergone to regulated intramembrane proteolysis (RIP) upon MTs binding, yielding soluble megalin intracellular domains (LRP-2-ICD; MICD) that are able to enter the nuclei and immediately modify gene expression [30,31,32]. This assumption is in line with previous in vitro findings of significantly altered expression of numerous proteins involved in energy homeostasis and cell survival after siRNA-mediated megalin knockdown in the melanoma cell lines [61]. It is worthy to mention again that initiation of the megalin RIP process is dependent on metalloproteinases [30,31,32] requiring a sufficient amount of zinc [41,42], wherein MTs pose the main physiological regulator of its bioavailability [4,5,6,7]. Such interrelationship underscores the value of considering the expression of both proteins together in an attempt of clarifying the impact of each one in the processes of oncogenesis and cancer progression, as well as when they are studied as potential biomarkers and predictors of disease outcome. The strong positive correlation between megalin and MTs expressions that we found in the overall studied group further indicates their functional link, but also allows speculation that megalin MICD domains, generated after MTs being ligated, induced additional megalin gene expression when they translocated in the nucleus, rendering a positive feedback loop, as has been shown in the case of the transthyretin (TTR) binding to the neuronal megalin [30]. Since megalin is a multiligand receptor mediating the internalization of many proteins and metabolites required for proper metabolic turnover [63], it is reasonable to assume that gaining of megalin expression might additionally contribute to the tumor progression by facilitating nutrients uptake by tumor cells. In this context, the indirect metabolic role of concomitantly expressed MTs should also be borne in mind since the activity of more than 300 metabolic enzymes depends on zinc [4,11], and MTs, as a pool of interchangeable zinc, can ensure its adequate supply during increased requirements [4,5,6,7]. This also applies to supporting the activities of zinc finger-containing transcription factors, some of which, such as ZEB1 and ZNF703, have been found to be human oncogenes responsible for pro-invasive and poorly differentiated stem-like cancer phenotype [66,67,68]. Interestingly, Ayinampudi and Narsimhan [69] found that patients with premalignant oral lesions and oral carcinoma had increased salivary zinc levels, and the increase showed the same pattern as we found for MTs and megalin induction in our samples. Furthermore, studies on animal models sowed zinc redistribution and accumulation at the sites of intensive cell proliferation and tissue growth [70].

Important and interesting detail having been demonstrated in this study is the striking megalin immunopositivity of chromosomes in the cells showing mitotic figures (Figure 3I,L; arrows). Such finding points to the possible immediate impact of megalin and/or cleaved MICD domains on the cellular division processes, and so far, chromosomal interactions with megalin or its soluble intracellular domain have not been reported. However, systemic developmental anomalies (faciooculoacousticorenal syndrome) arising from homozygous mutations of the LRP2/megalin gene [71,72] also suggest megalin involvement in the mitotic processes during development and growth. Here we have to emphasize that we used an anti-megalin antibody raised against C-terminal epitopes within amino acids 4411-4655 (H-245, Santa Cruz Biotechnology, Dallas, TX, USA) which constitute MICD domain [73], and considering RIP processing, there is a possibility of obtaining different staining patterns if antibodies being not directed against C-terminal fragments were to be used.

Another interesting observation that emerged from the study is the ability to clearly distinguish infiltrating cancer cells from healthy surrounding tissue at tumor edges in specimens having been stained with anti-MT I+II antibody (Figure 1M,N; arrows). This finding could be of practical importance when determining cancer invasion but have to be further confirmed.

Since cigarette smoking is widely considered as one of the major risk factors for oral mucosal pathology in general [46], and tobacco smoke ingredients, especially cadmium, have been found as being potent exogenous inducers of MTs expression [4,38], we compared the expression of MTs and megalin regarding to the smoking habits for each group of subjects separately. However, we did not find significant differences between smokers and non-smokers neither for MTs nor megalin staining intensities in any group (Figure 7). Similarly, we found no differences between males and females in any group (data not shown), although several studies on animal models reported that systemic MTs expression was sex-influenced and female-dominant, but with regard to the constitutive levels in the healthy tissues [74,75]. The absence of expression association with sex and smoking habits, as found in our study, emphasizes lesion-dependent upregulation of MTs and megalin.

In the present study we showed MTs-megalin interaction and their positive correlation in cancer tissue, but possible interactions of MTs with other molecules within the complex pro-survival and anti-apoptotic network should also be considered. Although investigations on that topic are scarce, several studies suggest potential interaction of MTs with survivin, which was found to show similar pattern of overexpression and localization in OSCC lesions [76], whereas simultaneous in vitro silencing of both survivin and MT-IIA expression showed more potent effects on cell proliferation in the aggressive ovarian tumor cell lines than either alone [77]. Together with our findings, these observations underline the importance of further research on the role of MTs interactions in the cancer pathogenesis and progression.

## 5. Conclusions

Overall, data obtained point to the onco-driving potential of interaction between MTs and megalin in the oral epithelium. The proposed intertwined interplay between these proteins gives rise to hypothesize the existence of the MTs-megalin functional axis, which can impact malignant transformation, tumor behavior, and its phenotype. On that trail, both molecules might pose promising therapeutic targets, as well as useful auxiliary tools for oral cancer staging and estimation of its invasion.

## Figures and Tables

**Table 1:** Basic demographic and clinicopathological features of involved subjects, and variables measured in the study.

	HEALTHY ORAL MUCOSA	ORAL LICHEN PLANUS	ORAL LEUKOPLAKIA	ORAL SQUAMOS CELL CANCER		
				GRADE I	GRADE II	GRADE III
N	10	15	26	28	23	12
SEX (M:F)	7:3	10:5	16:10	14:14	15:8	10:2
AGE AT DIAGNOSIS	51.1 ± 15.8	63.9 ± 9.8	61.4 ± 9.6	63.9 ± 8.4	65.4 ± 7	65.8 ± 10.6
SMOKING (Y:N)	6:4	4:11	13:13	16:12	13:10	5:7
MT—AVERAGE GRAY VALUE: MEAN±SD	12.0 ± 4.6	20.8 ± 4.9	28.4 ± 7.6	53.7 ± 12.4	85.2 ± 23.6	106.4 ± 25.4
MT—AVERAGE GRAY VALUE: MEDIAN (RANGE)	11.9 (5.3−18.4)	19.4 (14.2−31.1)	27.9 (17.8−46.9)	53.5 (21.7−93.7)	83.5 (52.2−137.6)	114.3 (67.2−133.6)
MEGALIN—AVERAGE GRAY VALUE: MEAN±SD	0	16.1 ± 8.8	25.6 ± 8.5	32.8 ± 7.8	44.1 ± 12.3	81.5 ± 13.4
MEGALIN—AVERAGE GRAY VALUE: MEDIAN (RANGE)	0	14.8 (6.1−35.0)	24.7 (9.6−43.2)	32.5 (7.1−51.5)	44.2 (20.4−68.3)	78.8 (64.6−105.4)

## Data Availability

The data presented in this study are available on request from the corresponding author if data sharing is approved by ethics committee. The data are not publicly available due to data protection laws and adherence to ethical principles.
